# Construction and Biomechanical Properties of PolyAxial Self-Locking Anatomical Plate Based on the Geometry of Distal Tibia 

**DOI:** 10.1155/2014/436325

**Published:** 2014-06-16

**Authors:** Weiguo Liang, Weixiong Ye, Dongping Ye, Ziqiang Zhou, Zhiguang Chen, Aiguo Li, Zong-Han Xie, Lihai Zhang, Jiake Xu

**Affiliations:** ^1^Guangzhou Institute of Traumatic Surgery, Guangzhou Red Cross Hospital, Medical College, Jinan University, Guangzhou 510220, China; ^2^School of Pathology and Laboratory Medicine, The University of Western Australia, Crawley, WA 6009, Australia; ^3^School of Mechanical Engineering, University of Adelaide, SA 5005, Australia; ^4^Department of Infrastructure Engineering, The University of Melbourne, VIC 3010, Australia

## Abstract

In order to provide scientific and empirical evidence for the clinical application of the polyaxial self-locking anatomical plate, 80 human tibias from healthy adults were scanned by spiral CT and their three-dimensional images were reconstructed using the surface shaded display (SSD) method. Firstly, based on the geometric data of distal tibia, a polyaxial self-locking anatomical plate for distal tibia was designed and constructed. Biomechanical tests were then performed by applying axial loading, 4-point bending, and axial torsion loading on the fracture fixation models of fresh cadaver tibias. Our results showed that variation in twisting angles of lateral tibia surface was found in various segments of the distal tibia. The polyaxial self-locking anatomical plate was constructed based on the geometry of the distal tibia. Compared to the conventional anatomical locking plate, the polyaxial self-locking anatomical plate of the distal tibia provides a better fit to the geometry of the distal tibia of the domestic population, and the insertion angle of locking screws can be regulated up to 30°. Collectively, this study assesses the geometry of the distal tibia and provides variable locking screw trajectory to improve screw-plate stability through the design of a polyaxial self-locking anatomical plate.

## 1. Introduction

The anatomical plate is an ideal internal fixation treatment for distal tibia fractures [1]. Since the distal tibia has a forward twisting transition anatomy, it is important to match the anatomical plate with the proper geometric structure of the distal tibia [2]. In order to provide anatomical information for the design of the anatomical plate of distal tibias for the domestic population in Southern China, it is of crucial importance to study morphologic characteristics of the distal tibia [3]. The distal tibia is close to the ankle joint with special anatomical morphology and a poor soft tissue envelope. Effective treatment of fractures in this special region remains a challenge to orthopaedic surgeons [4]. In recent years, the use of an anatomical locking plate for the treatment of distal tibia fractures has been widely reported with satisfactory results [5]. However, in the first generation of locking plates currently available, screw insertion angles are predetermined by manufacturers and are not able to be adjusted during instrumentation [6]. In order to make the plate better suited to the tibia geometry of Chinese people and to provide variable locking screw trajectory to improve screw-plate stability, we have designed a polyaxial self-locking anatomical plate for the distal tibia and performed biomechanical tests on cadaver fracture models.

## 2. Methods

### 2.1. Morphological Study of the Distal Tibia with Spiral CT

80 human tibia bones from healthy adults (38 male, aged from 20 to 70 years; 42 female, aged from 23 to 68 years) were scanned using spiral CT, and three-dimensional images were reconstructed using the surface shaded display (SSD) method. GE Hispeed ZX/i scanning was performed using the configuration shown in Table 1.

Three-dimensional reconstruction of tibia geometry was performed using a GE ADW4.0 Image workstation. Three anatomical indices (i.e., the length of twisting segment on the lateral surface of the tibia, twisting angles on different twisting segments, and the anteversion angle of the lateral surface of the tibia) were measured in the three-dimensional images by the GE ADW4.0 Image workstation. Because there was a different torsion angle on the outer surface of the tibia, in order to understand the torsion angle changes, we divided the twist section into four equal parts, that is, first twisting section, second twisting section, third twisting section, and fourth twisting section. The measured indices were then statistically analyzed using SPSS 12.0 statistical analysis software with T-test, ANOVA test and correlation analysis test. There was no history of trauma, surgery, or skeletal disorders in any of the individuals involved in this study.

### 2.2. Design of Polyaxial Self-Locking Anatomical Plate

The polyaxial self-locking anatomical plate for distal tibia was designed according to the morphologic characteristics of distal tibiae of the Chinese people. This design was granted a practical Patent by the Patent Bureau of China (number 201020525718.X). The plates are made from titanium alloy and were manufactured by Trauson Medical Instrument Company (Jiangsu, China).

### 2.3. Biomechanical Testing of the Polyaxial Self-Locking Anatomical Plate

Paired fresh cadaver tibiae were used to make fracture fixation models. Each pair of tibiae was examined radiographically to rule out any relevant pathological changes. Both ends of the tibia were potted in polymethylmethacrylate (PMMA) after explantation of soft tissues, during which normal saline was sprayed to keep the specimens moist. Each bone specimen was wrapped in a double layer plastic bag and stored at −20°C. Before instrumentation and biomechanical testing, each frozen bone was thawed at room temperature.

All paired cadaver tibiae from left and right tibiae were randomly distributed into two groups with six pairs in each group, and the conventional anatomical locking plates and polyaxial self-locking anatomical plates with 9 holes were applied in each group, respectively. For polyaxial self-locking anatomical plates, three locking screws were inserted into the three separated most proximal polyaxial holes on the plate to fix the proximal fracture segment while another three locking screws were inserted into the three most distal polyaxial holes to fix the distal fracture segment with the screw trajectories being regulated far from the fracture line. For conventional anatomical locking plates, six locking screws were inserted into the corresponding holes on the plate to fix the fracture segments, respectively. Osteotomies were performed to produce a highly unstable type-A fracture with the implant alone transferring all loads. The osteotomy levels were designed at the transition of segments 42 and 43 according to AO classification and 10 mm above it to create a 10 mm sized bone defect.

The biomechanical testing was conducted using the 858 Mini Bionix testing machine. For the axial loading test, a maximum of 500 N was loaded at a rate of 5 N/s on the point 10 mm mediodorsally to the intercondylar eminence. In 4-point bending test, a maximum of 300 N was loaded at a rate of 5 N/s with 4 cm loading distance and 12 cm pivot distance. To assess axial torsion loading, a maximum of 5 Nm was loaded at a rate of 0.1°/s. The constructs were preloaded to 10% of the maximum load before every test to rule out error caused by creep deformation.

### 2.4. Statistical Analysis of Data

Statistical analyses were performed using SPSS 12.0 statistical analysis software with *t*-test and ANOVA test.

## 3. Results

**Figure 1:**
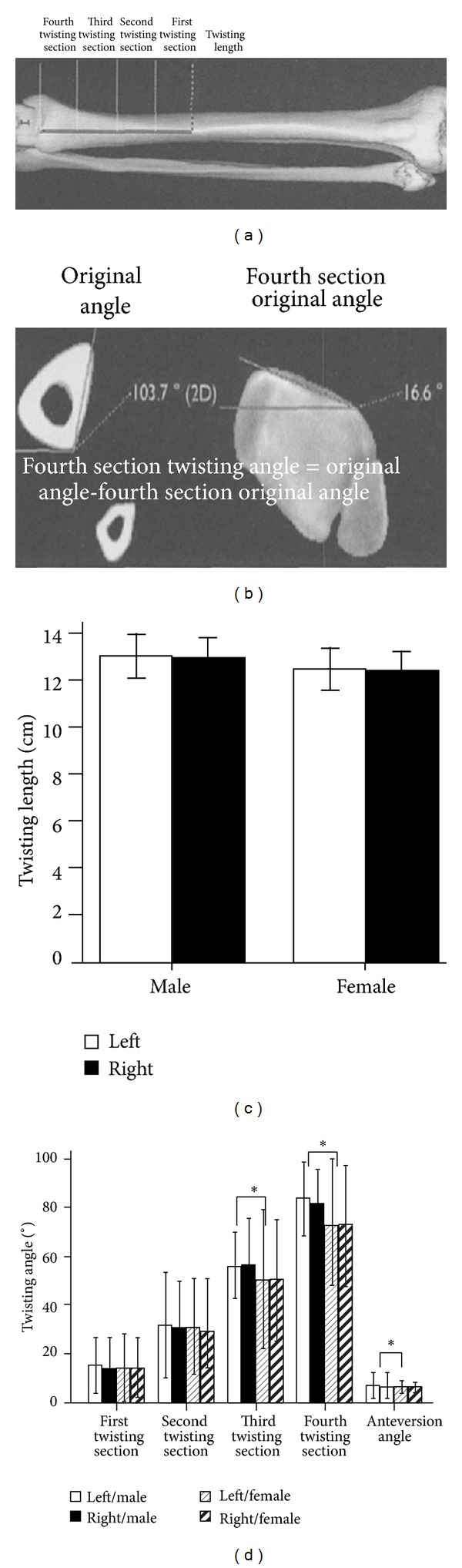
(a) The measurement of tibia anatomic lengths by spiral CT scanning. The torsion section was divided into four equal parts and the outer side torsion angle was measured individually in these four parts. (b) Measurement of the tibia twisting angle by CT scanner. (c) The length of the twisting segment of the lateral tibia surface, the twisting angle of the lateral surface of tibia in the first quarter, the second quarter, the third quarter, and the fourth quarter as well as the anteversion angle of the lateral surface of distal tibia were measured both in male and female individuals.

**Figure 2:**
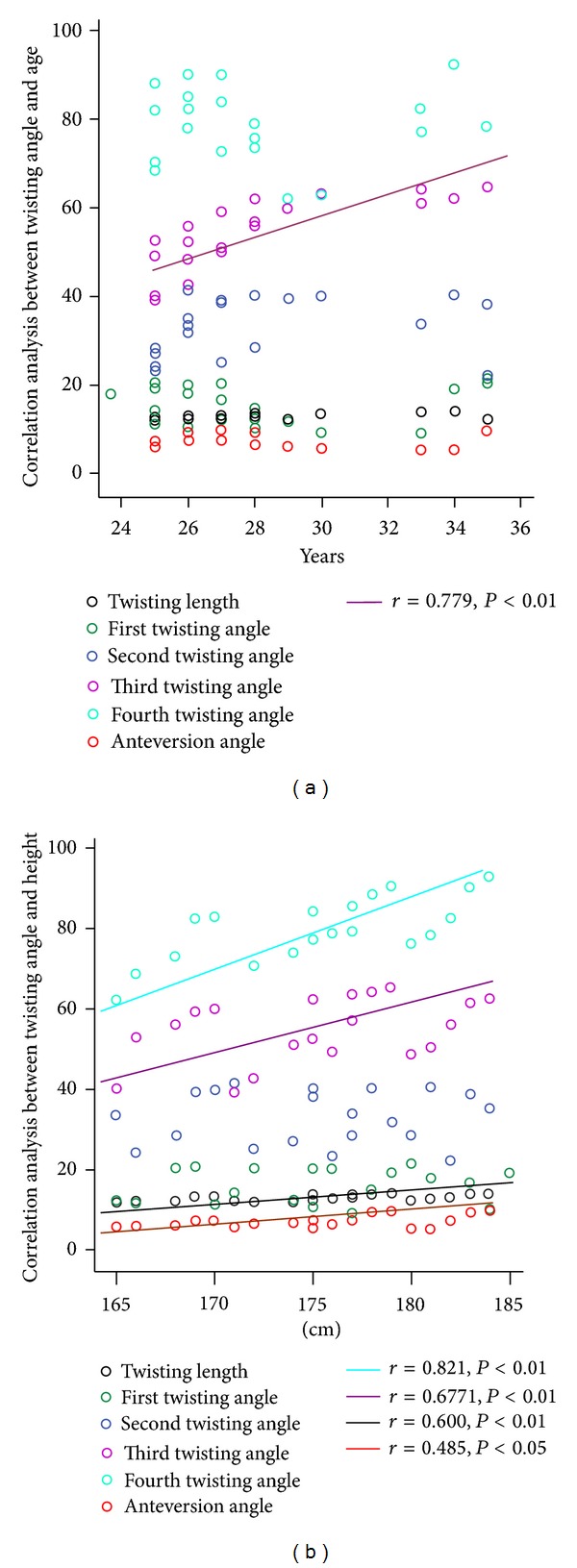
Correlation analyses between twisting angle and the age of individuals (a) and between twisting angle and the height of individuals (b).

In order to obtain precise geometrical measurement of the distal tibia in individuals in Southern China, 80 healthy adults were examined using three-dimensional spiral CT. Three parameters were collected as illustrated in Figure 1 including twisting length and twisting angles from the first, second, third and fourth quarters. The length of the twisting segment of the lateral tibia surface was determined to be 12.9 ± 0.41 cm in males and 12.34 ± 0.31 cm in females (Figure 1(c)). The twisting angle of the lateral surface of the tibia was determined to be as follows (Figure 1(d)): (1) in the first quarter, the twisting angle of the lateral tibia surface was 13.98 ± 2.72° in males and 13.38 ± 3.11° in females; (2) in the second quarter, it was 32.49 ± 3.66° in males and 31.85 ± 3.86° in females; (3) in the third quarter, it was 55.18 ± 3.53° in males and 50.95 ± 6.24° in females; and (4) in the fourth quarter, it was 82.13 ± 2.89° in males and 72.45 ± 4.81° in females. The anteversion angle of the lateral surface of the distal tibia was 7.34 ± 0.91° in males and 6.20 ± 0.41° in females, respectively (Figure 1(d)). Among all the measurements, the twisting angles of the third and fourth quarters and the anteversion angle were statistically significant between the males and females (*P* value <0.05) (Figure 1(d)). In addition, it can be seen that there is a strong correlation between the third twisting angle and the age of individuals (*P* value <0.01) (Figure 2(a)). Further, the twisting length, the 3rd and 4th twisting angles, and the anteversion angle were statistically significantly and correlated to the height of individuals with a *P* value <0.01; <0.01; <0.01; and <0.05, respectively (Figure 2(b)).

**Figure 3:**
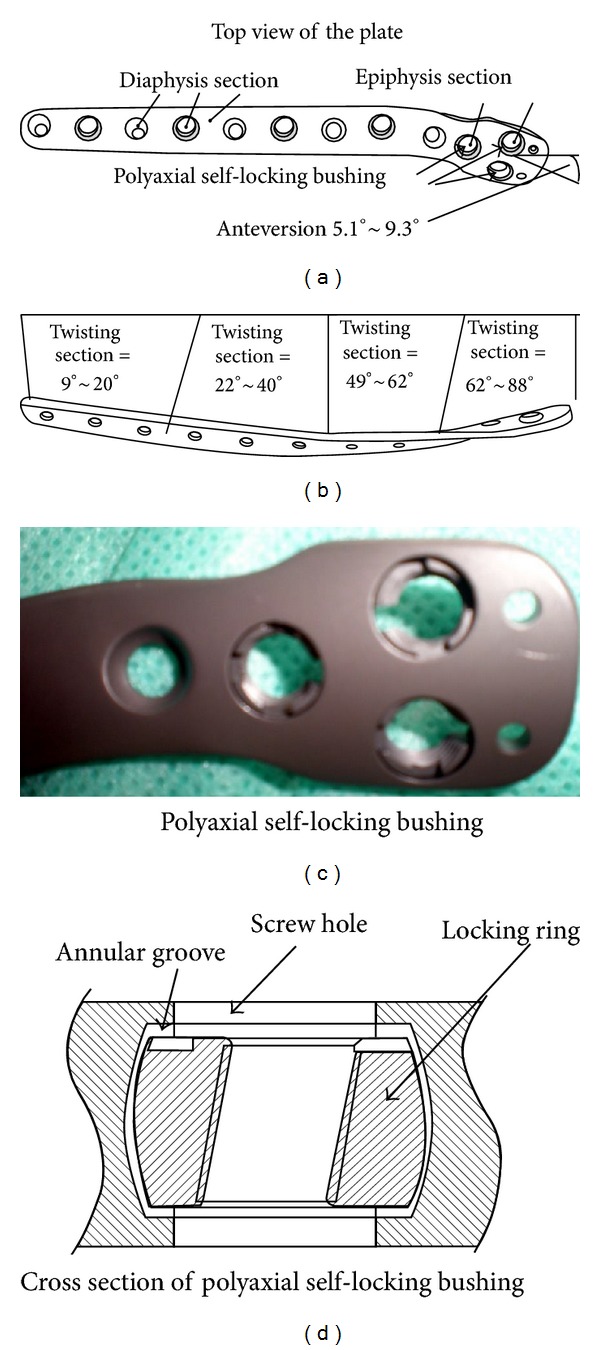
(a)-(b)Design of polyaxial self-locking anatomical plate, anteversion, 5.1°~9.3°; twisting section, 9°~20°; twisting section, 22°~40°; twisting section, 49°~62°; twisting section, 62°~88°. (c) The polyaxial self-locking bushing is situated within the polyaxial hole, which is round shaped from the top view with a C-shaped defect. On the obverse surface of the bushing, there are three triangularly distributed small concaves. (d) A cross sectional view of the polyaxial self-locking bushing shows that it is bucket-shaped with a polished outer surface and threaded inner surface.

Based on the data collected, the polyaxial self-locking anatomical plate was designed in a spoon-like shape with a flared distal part and a long-stem proximal segment (Figure 3). The distal segment of the plate twists anterior with the largest twisting angle being 80° and a 12 cm long twisting segment. Three polyaxial locking holes are located at distal part of the plate and are distributed triangularly. In the proximal stem part of the plate, polyaxial locking holes are distributed separately to each other with conventional anatomical locking holes. The top view of the polyaxial hole is a round shape whilst the cross section of the polyaxial hole is concave tympaniform, allowing the polyaxial self-locking bushing to be rotated laterally within it to a maximum of 5°.

The polyaxial self-locking bushing is situated within the polyaxial hole, which is round shaped from the top view with a C-shaped defect (Figure 3(c)). On the obverse surface of the bushing, there are three triangularly distributed small concaves which are separated 90° from the C-shaped defect. A cross sectional view of the polyaxial self-locking bushing shows that it is bucket-shaped with a polished outer surface and threaded inner surface. The inner axis is intersected with the outer axis at 10°. The polyaxial bushing is clasped by the upper and lower outlets of the polyaxial hole and fits precisely with the inner surface of the polyaxial hole. The C-shaped defect of the polyaxial bushing and the three concaves on its obverse surface fit precisely with the four dental processes on the tip of the polyaxial regulating sleeve which drives the rotation of polyaxial bushing in the polyaxial hole to regulate the locking angle of the screw. Once the locking screw is tightened, the polyaxial self-locking bushing is expanded and fits tightly with the concave surface of the polyaxial hole so that strong friction is produced, fixing the locking angle with the screw. The polyaxial bushing can be laterally rotated up to 5°, which together with the 10° eccentric angle of its inner thread axis can increase angular regulation amplitude for the locking screw up to 30°.

**Figure 4:**
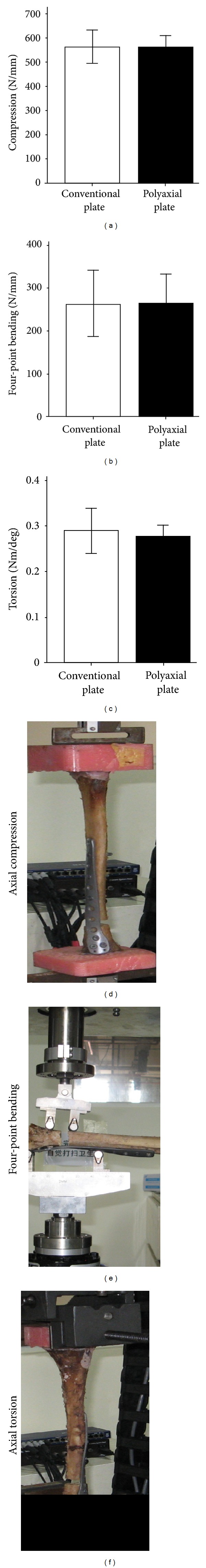
Biomechanical tests of the polyaxial self-locking anatomical plate. (a, d) Compression stiffness of conventional anatomical locking plate versus the polyaxial self-locking anatomical plate. (b, e) 4-point bending stiffness of conventional anatomical locking plate versus the polyaxial self-locking anatomical plate. (c, f) Torsion stiffness of conventional anatomical plate versus the polyaxial self-locking anatomical plate. Note that there is no statistical significance in all three tests.

The polyaxial self-locking anatomical plate provides a better fit to the geometry of the distal tibia when compared with the conventional anatomical locking plate as it can increase the angular regulation amplitude for the locking screw up to 30°. However, there was no statistical significance in the mechanical properties between the polyaxial self-locking anatomical plate and the conventional anatomical locking plate. For example, compression stiffness of the conventional anatomical locking plate was 557.53 ± 20.72 N/mm, and the polyaxial self-locking anatomical plate was 562.80 ± 28.26 N/mm (Figures 4(a) and 4(d). The 4-point bending stiffness of the conventional anatomical locking plate was 268.02 ± 36.77 N/mm, and the polyaxial self-locking anatomical plate was 265.76 ± 27.21 N/mm (Figures 4(b) and 4(e)). The torsion stiffness of the conventional anatomical locking plate was 0.28 ± 0.01 Nm/deg and that of the polyaxial self-locking anatomical plate was 0.29 ± 0.02 Nm/deg (Figures 4(c) and 4(f)).

## 4. Discussion

Anatomical locking plates have been successfully used in the treatment of a large number of metaphyseal fractures [7–9]. The anatomical locking plate for the distal tibia was developed to accommodate the need for the biological osteosynthesis of distal tibia fractures, which can effectively preserve the blood supply of the fracture site by MIPPO (minimally invasive percutaneous plate osteosynthesis) technique, therefore reducing the incidence of delay union, nonunion, and soft tissue complications [10]. However, the screw insertion trajectories of the first generation locking plates are predetermined by manufacturers and are not able to be regulated during instrumentation, thus limiting the plates' clinical application. In certain fracture patterns even if the screws were located at the fracture site, the bone defect area, or not the main fracture fragment, the screw insertion trajectory could not be changed, thereby severely affecting the fixation and even leading to failure [11].

In the present study, we measured the geometry of the lateral distal tibiae in 80 healthy adults using a three-dimension spiral CT scanner. According to parameters obtained in the study, the maximal twisting angle for the polyaxial plate was determined to be 80° and the twisting segment was 12 cm. With the regulation of polyaxial bushing in the plate hole by an angular regulation sleeve, the locking angle of the screw can be regulated as much as 30° which allows the surgeon to insert the locking screw more desirably. It is important to note that in order to design the polyaxial self-locking anatomical plate with optimal twist angle and anteversion, the difference in gender, age, and height of a patient must be considered.

We also compared the biomechanical properties of the polyaxial self-locking anatomical plate with those of a conventional anatomical locking plate and provided scientific evidence for its clinical application. A relatively high transverse defect osteotomy, 10-mm-wide, at the transition of diaphysis and metaphysis was chosen as a fracture model. This model mimicked a highly unstable metaphyseal fracture, with the implant alone transferring all loads between the two fracture fragments. A limitation of this model is that the defined defect, which prevents interlocking of the bony fragments during biomechanical testing, may influence the test results.

The tibia torsion angle was first defined as the connection between the horizontal line of the proximal tibia's articular surface and the coronal line of the distal tibia's articular surface by Tuttle and Manley [12]. In order to design the polyaxial self-locking anatomical plate, the outer side of the distal tibia torsion angle must be considered [13, 14]. In this study, we found that the outer side of the distal tibia torsion angle was different from that found in previous studies [15, 16]. The torsion segment length is from where the outer side of the tibia begins to twist to the front of distal tibia's articular surface [17–19]. To determine the torsion change of the transition section, we divided the torsion section into four equal parts and measured the outer side torsional angle for each part individually [20–22]. It was also observed that when the outer side of the middle and distal tibia twist from the sagittal plane to the coronal plane, its longitudinal axis has a little forward offset, which we define as the outer side tibia anteversion. One limitation of this study has been the relatively small number of experimental samples to determine Chinese people's distal tibial plate parameters, considering that Chinese people might have a large stature gap across different regions. Nevertheless, the results should be a valuable reference for future clinical studies.

Taken together, this study provides geometrical data on the distal tibias of Chinese people and constructs a variable locking screw trajectory to improve screw-plate stability by using the polyaxial self-locking anatomical plate of the distal tibia.

## Figures and Tables

**Table 1:** GE Hispeed ZX/i scanner configuration used in this study.

Parameter	Value
Position	Full-length of tibia
Method	Through horizontal surface
Layer distance	2 mm
Show vision	204
Tube voltage	120 kV
Elective current	240 mA

## References

[1] Kloc PA, Kowaleski MP, Litsky AS, Brown NO, Johnson KA (2009). Biomechanical comparison of two alternative tibial plateau leveling osteotomy plates with the original standard in an axially loaded gap model: an in vitro study. *Veterinary Surgery*.

[2] Hellio Le Graverand M-P, Buck RJ, Wyman BT (2009). Subregional femorotibial cartilage morphology in women—comparison between healthy controls and participants with different grades of radiographic knee osteoarthritis. *Osteoarthritis and Cartilage*.

[3] McCann L, Ingham E, Jin Z, Fisher J (2009). An investigation of the effect of conformity of knee hemiarthroplasty designs on contact stress, friction and degeneration of articular cartilage: a tribological study. *Journal of Biomechanics*.

[4] Fatone S, Gard SA, Malas BS (2009). Effect of ankle-foot orthosis alignment and foot-plate length on the gait of adults with poststroke hemiplegia. *Archives of Physical Medicine and Rehabilitation*.

[5] Lee JH, Dyke JP, Ballon D, Ciombor DM, Rosenwasser MP, Aaron RK (2009). Subchondral fluid dynamics in a model of osteoarthritis: use of dynamic contrast-enhanced magnetic resonance imaging. *Osteoarthritis and Cartilage*.

[6] Wong AKO, Beattie KA, Emond PD (2009). Quantitative analysis of subchondral sclerosis of the tibia by bone texture parameters in knee radiographs: site-specific relationships with joint space width. *Osteoarthritis and Cartilage*.

[7] Ronga M, Shanmugam C, Longo UG, Oliva F, Maffulli N (2009). Minimally invasive osteosynthesis of distal tibial fractures using locking plates. *Orthopedic Clinics of North America*.

[8] Cheng Z, Zhang F, He F (2010). Osseointegration of titanium implants with a roughened surface containing hydride ion in a rabbit model. *Oral Surgery, Oral Medicine, Oral Pathology, Oral Radiology and Endodontology*.

[9] Rapuri VR, Clarke HD, Spangehl MJ, Beauchamp CP (2011). Five cases of failure of the tibial polyethylene insert locking mechanism in one design of constrained knee arthroplasty. *The Journal of Arthroplasty*.

[10] Spiegelberg BGI, Sewell MD, Aston WJS (2009). The early results of joint-sparing proximal tibial replacement for primary bone tumours, using extracortical plate fixation. *Journal of Bone and Joint Surgery B*.

[11] Bordelon JT, Coker D, Payton M, Rochat M (2009). An in vitro mechanical comparison of tibial plateau levelling osteotomy plates. *Veterinary and Comparative Orthopaedics and Traumatology*.

[12] Tuttle TA, Manley PA (2009). Risk factors associated with fibular fracture after tibial plateau leveling osteotomy. *Veterinary Surgery*.

[13] Ronga M, Longo UG, Maffulli N (2010). Minimally invasive locked plating of distal tibia fractures is safe and effective. *Clinical Orthopaedics and Related Research*.

[14] Hayes JS, Seidenglanz U, Pearce AI, Pearce SG, Archer CW, Richards RG (2010). Surface polishing positively influences ease of plate and screw removal. *European Cells and Materials*.

[15] Gessmann J, Seybold D, Baecker H, Muhr G, Graf M (2009). Correction of supramalleolar deformities with the Taylor spatial frame. *Zeitschrift für Orthopädie und Unfallchirurgie*.

[16] Hoenig M, Gao F, Kinder J, Zhang LQ, Collinge C, Merk BR (2010). Extra-articular distal tibia fractures: a mechanical evaluation of 4 different treatment methods. *Journal of Orthopaedic Trauma*.

[17] Oh JK, Sahu D, Hwang JH, Cho JW, Oh CW (2010). Technical pitfall while reducing the mismatch between LCP PLT and upper end tibia in proximal tibia fractures. *Archives of Orthopaedic and Trauma Surgery*.

[18] Apelt D, Pozzi A, Marcellin-Little DJ, Kowaleski MP (2010). Effect of cranial tibial closing wedge angle on tibial subluxation: an ex vivo study. *Veterinary Surgery*.

[19] Conkling AL, Fagin B, Daye RM (2010). Comparison of tibial plateau angle changes after tibial plateau leveling osteotomy fixation with conventional or locking screw technology. *Veterinary Surgery*.

[20] LaPrade RF, Oro FB, Ziegler CG, Wijdicks CA, Walsh MP (2010). Patellar height and tibial slope after opening-wedge proximal tibial osteotomy: a prospective study. *The American Journal of Sports Medicine*.

[21] Fitzpatrick N, Johnson J, Hayashi K, Girling S, Yeadon R (2010). Tibial plateau leveling and medial opening crescentic osteotomy for treatment of cranial cruciate ligament rupture in dogs with tibia vara. *Veterinary Surgery*.

[22] DeMeo PJ, Johnson EM, Chiang PP, Flamm AM, Miller MC (2010). Midterm follow-up of opening-wedge high tibial osteotomy. *The American Journal of Sports Medicine*.

